# Willingness of WHO staff to work in health emergencies in the African Region: opportunity for phased deployment of staff and ensure continuity of health services

**DOI:** 10.11604/pamj.2024.47.68.40362

**Published:** 2024-02-16

**Authors:** Abdou Salam Gueye, Joseph Okeibunor, Reuben Ngofa, Ishata Conteh, Nkechi Onyeneho, Nodjilembaye Mbainodji, Fiona Braka, Dick Chamla, Etien Luc Koua, Matshidiso Moeti

**Affiliations:** 1 WHO African Region, Brazzaville, Congo,; 2 University of Nigeria, Nsukka, Nigeria

**Keywords:** Emergencies, health workers, WHO staff, African Region, AVoHC surge

## Abstract

A human resource base that ensures appropriate deployment of staff to emergencies, addressing different shock events in emergencies, without disrupting continuity of service is germane to a successful response. Consequently, the WHO Health Emergencies programme in the African Region, in collaboration with Africa Centre for Disease Control (ACDC) launched the African Volunteer Health Corps (AVoHC) and Strengthening and Utilization of Response Group for Emergencies (SURGE), an initiative aimed at ensuring a pool of timely responders. We explored the willingness of WHO staff to work in emergencies. A call for expression of interest to be part of the Elite Emergency Experts (Triple E) was published on 5^th^ July 2022 via email and was open for 5 weeks. The responses were analyzed using simple descriptive statistics and presented with graphic illustrations. A total of 1253 WHO staff, from all the six WHO regions, cutting across all cadre, applied to the call. The applicants had various trainings and experiences in emergency and have responded to mostly disease outbreaks. Two-third of the applicants were males. This paper did not explore reasons for the willingness to work in emergencies. However, contrary to fears expressed in literature that health workers would not want to work in emergencies with potential for infections, the applicants have worked mostly in infectious emergencies. Literature identified some themes on factors that could impact on willingness of health workers to work in emergencies. These include concerns for the safety of the responders and impact of partners, child and elderly care, as well as other family obligations, which emergency planners must consider in planning emergency response.

## Introduction

The Africa region witnesses numerous public health emergencies from diverse pathogens and often in challenging settings including complex humanitarian emergencies every year. In last decade the region has recorded over 100 public health events (PHEs) annually [1]. Approximately 80% of the reported events are emerging and re-emerging infectious diseases [2]. These events have significant implications for global health security and universal health coverage gains [3], and are often associated with high morbidity and mortality as well as significant socio-economic disruptions. Despite advances in the countermeasures to combat these events, the unpredictability of their occurrence and impacts make them challenging to contain, with potential to significantly threaten both global health security (GHS) and progress towards achieving universal health coverage on the region [3]. Often, emergency planners and managers and even the public presume emergency health workers, including staff of the World Health Organization (WHO), will be willing to work. These categories of health workers may be reluctant to work when the situation poses a possible threat to their own safety and health [4]. Studies have shown that the willingness of emergency health care workers to report for work during major emergencies is influenced by perception of personal risk and fear for safety of family members [5]. For instance, the infectiousness of SARS in 2003 was substantially higher among health care workers than the general population, especially those working in hospitals and prehospital care [6].

Health workers involved in the medical care of SARS patients showed some reluctance because of their concerns about their own health and the health of their family, and developed fear of social contact [7,8]. Health care workers believed that they were at high risk of becoming infected, with some refusing to care for the ill and imposing self-quarantine on themselves to protect family members from potential exposure [9]. These behaviours are reminiscent of the psychosocial reactions witnessed in Ebola Virus Disease (EVD) outbreaks following the West Africa EVD outbreak of 2014-2016, where a total of 881 health workers were infected and 513 died, while caring for patients, in Guinea, Liberia and Sierra Leone [10,11]. This engenders significant fear that results in reluctance, which may pose a challenge to the ability of the health system to cope with the surge in demand for resources commensurate to the magnitude of the emergency event [7,8,12]. This may explain the persistent gap in ability of emergency planners to promptly deploy skills in line with shocks and phases of different emergency event in the African Region, despite many years of responding to these emergencies. There is need for a human resource base that would permit appropriate deployment of staff to respond to the different shock events in emergencies, which introduce new services not envisaged or planned for, such as caring for Ebola victims during an outbreak [13].

Following from the foregoing, the African Volunteer Health Corps and Strengthening and Utilization of Response Group for Emergency (AVoHC-SURGE), an initiative birthed out of the close collaboration between African Center for Disease Control, World Health Organization Regional Office for Africa, and African Member States was launched. AVoHC aims at speedy deployment of emergency responders in all public health facets, while limiting the disruption of essential health services, minimizing morbidity, and reducing mortality through a pool of national-level cadres of local human resources with the technical, operational, and logistical know-how to respond to health emergencies and humanitarian crises. As part of the regional workforce development, happening across the African Region, WHO AFRO established an internal roster of experts amongst its staff, trained, and ready to be speedily deployed, in support of the already existing in-country AVoHC-SURGE health work force. Given that a willing and able emergency health care workforce will be a vital component of any successful response to an emergency event, clarity on the willingness of WHO staff to work and barriers to willingness to work during major emergencies is necessary for emergency planning and response. This paper analyses response to the WHO health emergency preparedness and response (WHO/EPR) programme AVoHC-SURGE call on WHO staff interested to be listed on the rooster of potential responders in major emergencies. This paper therefore explores and assesses the willingness of WHO staff to work in health emergencies in the African Region.

## Program Assessment

### Methods

**Figure 1:**
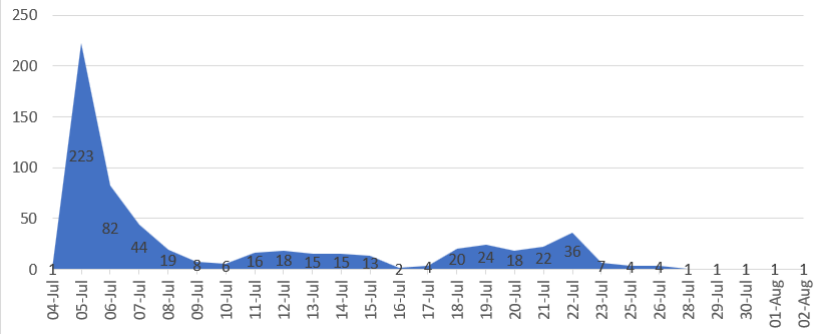
submission timestamp trend

**Triple-E selection process:** the Call for Expression of Interest to be part of the Elite Emergency Experts (Triple E) was published and circulated widely on 5^th^ July 2022 by the head of human resource via email. This was open for 5 weeks after publication (Figure 1). A link was included in the email circulated. This link gives access to the online questionnaire for the expression on interest.

### Technologies used

***Enketo Forms:*** leveraging on the Polio legacy and resources, an online questionnaire to collate the expressions of interest was developed in three languages (English, French, and Portuguese) using the Enketo - A Polio owned premise-based server developed and deployed for Polio surveillance activities. A web access link of the online questionnaire was generated which was included in the email circulated to give interested persons access to express their interest(s).

***Text It:*** this is a dynamic and robust tool that allows anyone to visually build interactive SMS and Voice applications anywhere in the world using its exclusive flow engine. In this context, the Text It flow was integrated into the Enketo form that triggers an already scripted automated message that notifies the responders once their submission is successful.

**Figure 2:**
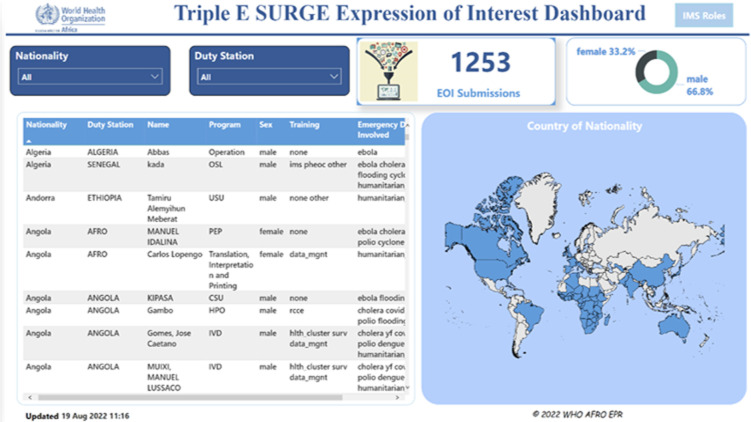
screenshot of automated dashboard visualizing a real-time data submission

***Power BI:*** using Microsoft Business Intelligence tool, a live dashboard was developed to monitor the data submission in real time. An API for the Enketo form was developed and plugged into the PowerBI backend to stream-in data directly in real-time (Figure 2).

## Results

### Demographic and emergency profiles of the applicants

**Figure 3:**
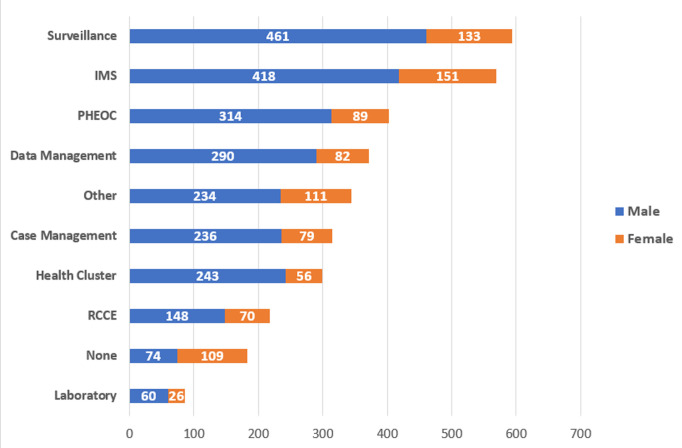
distribution of applicants by training on emergency received and gender

A total of 1253 expressions of interests were received. Two-thirds (67.0%) of the applicants were male. The applicants fell into different categories of demographic groupings and experiences in emergency operations. The applicants are drawn from all grade levels of UN employment, ranging from National Officers to interns and gradeless staff (Table 1). The contract types of the applicants include UN volunteers (33.4%), staff on temporary appointments (21.6%) and staff of fixed term appointment (8.1%) among others. The applicants are spread across the six WHO regions, with a majority from WHO African Region (96.01%). Within the African Region, the applicants are spread across the four subregions and the Regional Office for Africa (AFRO). The majority were from West Africa (31.0%) and Eastern Africa (29.0%). The regional office based in Brazzaville constituted 19.0 percent of the applicants from the African Region. In terms of the emergency experience, Table 1 further revealed that the commonest emergency area, where the applicants have received training were surveillance (47.0%); incident management system (IMS) (45.0%). The least area of expertise was laboratory (7.0%) and risk communication and community engagement (RCCE) (17.0%). Emergency deployment pattern revealed that the greatest majority had been deployed to COVID-19 response (72.0%). Other emergencies with high mention by the applicants include cholera (53.0%); polio (50.0%) and measles outbreak (46.0%). Small proportions of the applicants have engaged in non-infectious emergencies like flooding (22.0%); cyclone (8.0%); and mudslide (6.0%). Figure 3 revealed gender differentials with respect to trainings in emergency programmes the applicants had participated in. In all, the males dominated. For instance, on surveillance, there were 461 males compared to the 133 females. Similarly, for training in incident management system (IMS), there were 418 males compared to 151 females.

**Table 1:** distribution of all applicants by demographic and experience in emergency operations groupings (N=1,253)

Grouping	Category	Number	Percentage
Gender	Male	837	67
	Female	416	33
Contract Type	Short Service Agreement	418	3.8
	Fixed Term Appointment	271	21.6
	Continuous Appointment	233	18.6
	Consultant	179	14.3
	Temporary Appointment	102	8.1
	UN Volunteer	48	3.8
	Internship	2	0.2
WHO Region of Origin	Africa	1203	96.01
	Europe	18	1.44
	Americas	14	1.12
	Eastern Mediterranean	11	0.88
	Western Pacific	4	0.32
	Southeast Asia	3	0.24
AFR Duty Station	Western Africa	392	31
	Eastern Africa	361	29
	AFRO	232	19
	Central Africa	215	17
	Southern Africa	53	4
Training in Emergencies Done	Surveillance	594	47
	IMS	569	45
	PHEOC	403	32
	Data Management	372	30
	Case Management	315	25
	Health Cluster	299	24
	RCCE	218	17
	Laboratory	86	7
	Other	345	28
	None	183	15
Emergency Deployment	COVID-19	900	72
	Cholera	660	53
	Polio	628	50
	Measles	577	46
	Ebola	535	43
	Humanitarian	442	35
	Yellow Fever	428	34
	Flooding	278	22
	Lassa Fever	245	20
	Cyclone	102	8
	Dengue	100	8
	Mudslide	6	6
	Other	170	14
	None	60	5

**Figure 4:**
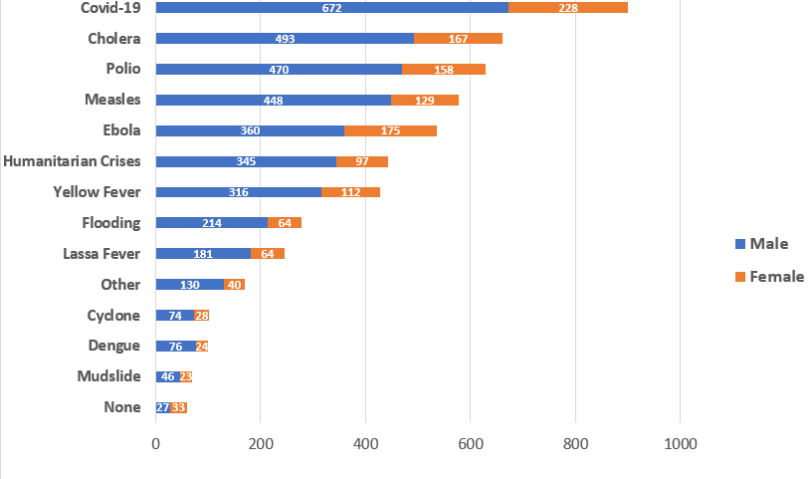
distribution of applicants by emergency deployment and gender

**Figure 5:**
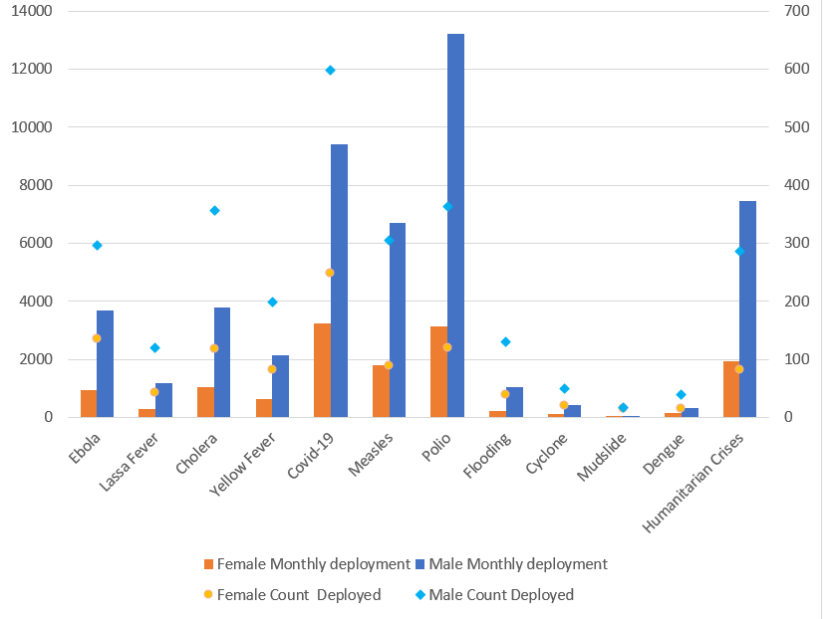
monthly deployment by gender

Figure 4 also revealed gender differentials on deployment patterns like the picture on the trainings. The number of males deployed to COVID-19 response were thrice in number (672) compared to the females (228). This is the case with all the emergencies for which they applicants have ever been deployed. The monthly deployment record in Figure 5 revealed picture like those in Figure 3 and Figure 4. Across every health emergency, the number of men deployed more than doubled the number of women on a month-to-month analysis. For instance, while less than 3000 women were deployed for Ebola response, there were close to 6000 men deployed for the same Ebola outbreak on monthly basis. With respect to COVID-19 response, there were about 5000 women deployed monthly compared to 12000 men for the same month. However, for emergencies like mudslide, there were nearly equal number of males and female responders. Table 2 focused on core WHO staff in the three categories of General Staff, National Professional Officer and Professional Officer, numbering 2,424 in the region. A quarter (606) of these responded to the call. With respect to gender of the WHO staff who applied, 28% of the females and 23% of the males applied (P < 0.01). in terms of contract type, more of the National Professional Officers (27%) then the General Service Staff (25%) and Professional Staff (23%) applied. However, the difference here was not statistically significant (>0.32). Table 2 also revealed that more staff in duty stations in the sub-regions than staff in the Regional Office in Brazzaville applied (p < 0.001). It also revealed that staff in the Eastern African (34%) and Central African (34%) sub-regions applied. Southern African sub-region had the least application from among the WHO staff.

**Table 2:** distribution of WHO AFRO staff that applied by demographic characteristics (N=2,424)

Groupings	Categories	Number	% Applied
Gender	Male	1608	376 (23%)
	Female	816	230 (28%)
Contract Type	General Service Staff National Professional	1122	279 (25%)
	Officer	654	176 (27%)
	Professional Officer	648	151 (23%)
AFR Duty Station	Western Africa	716	171 (24%)
	Eastern Africa	442	148 (34%)
	AFRO	640	145 (23%)
	Central Africa Southern Africa	318 331	108 (34%) 34 (10%)

## Discussion

The analysis of the response to the WHO Regional office for Africa (AFRO) and African Centre for Disease Control (ACDC) AVoHC-SURGE revealed great willingness to work in emergencies, among staff members of the World Health Organization, drawn from across the six regions of the world, including different categories of career path and expertise. Within a five-week period, the call received a whooping 1253 responses. This contradicts the conclusions drawn from a published review of literature, which held that the assumption that emergency health care workers will be willing to work during major emergencies and disasters is not a realistic one. In the conclusion of literature review, it was argued that willingness to work is “higher for ´conventional´ (physical) disasters, where infection and contamination of care providers are less likely to be issues, than for ´nonconventional´ (disease related) disasters, where responders are at risk of becoming infected, ill, and may possibly in turn expose family members to infectious or contaminated agents” [4]. The major factor, here, is the protection of emergency responders from becoming victims themselves by being infected. On the contrary, those who expressed willingness to work in emergencies have worked in African Region with numerous public health emergencies, mostly from diverse pathogens, some of which are highly infectious and often in challenging settings including complex humanitarian emergencies. It bears reiteration to mention that the region reports over 100 public health events (PHEs) annually1, out of which ~80% are emerging and re-emerging infectious diseases [2]. Thus, this high response to the AVoHC-SURGE reflects of WHO staff willingness to work even infectious events in Africa, contrary to pessimisms expressed in literature.

In this analysis, the reasons for the willingness to work in emergencies in the region were not explored. However, the responders were requested to indicate health emergency situations in which they had experiences and COVID-19 topped the list with 72% mentions out of 1253 response. Cholera, Polio, Measles and Ebola came next with 53%, 50%, 46% and 43% respectively. On the other hand, the conventional noninfectious emergencies like Cyclone and Mudslide had only 8% and 6% mentions respectively. All the same, it could be argued that given the preponderance of the applicants from the African Region (96.01%), where the conventional emergencies are rare, the applicants may not have had the opportunity to respond to these conventional emergencies. All the same, of importance to emergency planners should be the reported concerns for safety of the responders at emergencies. Previous studies indicate that 86% of the Israeli hospital personnel surveyed reported that their willingness to work during emergency situations would increase if they were provided with adequate “safety measures” and “protective equipment” [14]. Koh *et al*. noted a similar result in the SARS outbreak in Singapore, with a greater number of health care workers being willing to work during a similar event if further safety measures, namely protective equipment and education, among others, were available to staff [8].

Other recurring themes from past studies, which emergency planners must be mindful of include the impact of partner, childcare, and eldercare obligations. According to Smith, the desire for health care workers to provide care and reassurance to family members should be recognised and addressed in emergency preparedness plans [4]. The inability to fulfil these obligations may have a profound influence on willingness to report to work. Qureshi *et al*. emphasized the need for emergency response planners to pre-plan the formation of emergency childcare and eldercare facilities that can be either on or off-site, or by facilitating the pre-planned formation of emergency childcare/eldercare “pools”, where health care workers can leave their family members in the custody of people that they already know and trust [15]. Fears for personal safety and personal health issues were commonly flagged and should be addressed. These concerns for individual and family protection may also explain the low representation of the female staff in the 1253 applications received in response to the AVoHC call. Only a third of the number comprised of female staff of the WHO. It could be argued, however, that the sex ratio of 201.2 of the applicants reflects the reality of the actual population of staff in the WHO. Achieving gender parity in the UN staffing has remained a concern of UN organizations like WHO for a long time, with huge male dominance [16]. All the same, the low number of female applicants could also be due to concerns of domestic demands on the womenfolk, who are traditional care givers and producers of household health in Africa [17]. Qureshi *et al*. have elaborated on the impact of partner, childcare, and eldercare obligations in the household as a factor in willingness to work in health emergencies. Thus, these household obligations rather than the sex ratio in WHO staffing may provide better explanation for the number of females in WHO willing to work in health emergencies in the African Region. It is also a critical point of note for emergency response planners if the number of female responders could be enhanced. Gender sensitive response has been flagged as germane to humanitarian emergencies with different impacts on men, women, girls, boys and persons of all genders [18]. It is often stressed that people have different risks in emergencies, due to their sex and gender, which if not recognized could pose significant challenge to uptake of interventions.

## Conclusion

This paper demonstrates that high level of willingness among WHO staff to work in emergencies in the Africa Region, irrespective of being contagious or not. This is good for emergency planning. It is commonplace knowledge that the willingness to work decreased the longer that an event lasts, as was the case with the 2014-2016 Ebola Virus Disease outbreak in West Africa and even the recent COVID-19 pandemic. In the event for long and protracted events there is need for a human resource base that would permit appropriate deployment of staff to respond to the different shock events in emergencies, which introduce new services not envisaged or planned for, such as caring for victims during an outbreak. Another point of conclusion is that while this analysis could not elaborate on reasons for the willingness, in a review of past publications have flagged themes that must be addressed in emergency response planning. Some of the themes include safety of the responders and their protection against the emergency. Another theme is the concerns for partners, child and elder care, all of which may affect the willingness of females to work in emergencies, given the patriarchal nature of African society where women roles are detected by ethno-religious ideologies, economic as well as socio-cultural factors.
